# Factors associated with enteral nutrition tolerance after trauma laparotomy of the small bowel and mesenteric injuries by blunt trauma

**DOI:** 10.1186/s12893-023-01955-2

**Published:** 2023-03-23

**Authors:** Hyunseok Jang, Sangyun An, Naa Lee, Euisung Jeong, Yunchul Park, Jungchul Kim, Younggoun Jo

**Affiliations:** 1 Division of Trauma, Department of Surgery, Chonnam National University Medical School and Hospital, 42 Jebong-ro, Dong-gu, 61469 Gwangju, Republic of Korea; 2 Department of Surgery, Chonnam National University Medical School and Hospital, Gwangju, Republic of Korea

**Keywords:** Enteral nutrition, Mutiple trauma, laparotomy, Ileus, Intestine, small

## Abstract

**Background:**

In patients with blunt injury due to abdominal trauma, the common cause for laparotomy is damage to the small bowel and mesentery. Recently, postoperative early enteral nutrition (EEN) has been recommended for abdominal surgery. However, EEN in patients with blunt bowel and/or mesenteric injury (BBMI) has not been established. Therefore, this study aimed to identify the factors that affect early postoperative small bowel obstruction (EPSBO) and the date of tolerance to solid food and defecation (SF + D) after surgery in patients with BBMI.

**Methods:**

We retrospectively reviewed patients who underwent laparotomy for BBMI at a single regional trauma center between January 2013 and July 2021. A total of 257 patients were included to analyze the factors associated with enteral nutrition tolerance in patients with EPSBO and the postoperative day of tolerance to SF + D.

**Results:**

The incidence of EPSBO in patients with BBMI was affected by male sex, small bowel organ injury scale (OIS) score, mesentery OIS score, amount of crystalloid, blood transfusion, and postoperative drain removal date. The higher the mesentery OIS score, the higher was the EPSBO incidence, whereas the small bowel OIS did not increase the incidence of EPSBO. The amount of crystalloid infused within 24 h; the amount of packed red blood cells, fresh frozen plasma, and platelet concentrate transfused; the time of drain removal; Injury Severity Score; and extremity abbreviated injury scale (AIS) score were correlated with the day of tolerance to SF + D. Multivariate analysis between the EPSBO and non-EPSBO groups identified mesentery and small bowel OIS scores as the factors related to EPSBO.

**Conclusion:**

Mesenteric injury has a greater impact on EPSBO than small bowel injury. Further research is needed to determine whether the mesentery OIS score should be considered during EEN in patients with BBMI. The amount of crystalloid infused and transfused blood components within 24 h, time of drain removal, injury severity score, and extremity AIS score are related to the postoperative day on which patients can tolerate SF + D.

## Background

Among patients with abdominal trauma, blunt bowel and/or mesenteric injury (BBMI) accounts for approximately 5% of all abdominal injuries and is the third most common cause [1, 2]. Although traumatic injuries due to blunt trauma do not account for a large proportion worldwide, the proportion in South Korea is relatively high, reaching over 70% [3, 4].

BBMI is caused by direct force due to compression; collision with the abdomen affects the bowel itself and the mesentery, which is located between the abdominal wall and the retroperitoneal organs [5, 6]. Injuries caused by blunt trauma are often difficult to diagnose, and for patients who require immediate surgical treatment, the ranges of treatment and surgery for abdominal surgery are also diverse [7–9].

Similar to other abdominal surgeries, the initiation of enteral nutrition administration after abdominal surgery for patients with trauma is crucial for recovery. It has been reported that early enteral nutrition (EEN) can reduce postoperative morbidity and, by shortening the hospital stay period, can also reduce unnecessary medical expenses and resource use [10–12]. In addition, as a key component of the recent concept of enhanced recovery after surgery (ERAS), the rapid initiation of EEN has been emphasized and widely implemented [13]. In particular, in cases of abdominal surgery, recent studies have proven that EEN is feasible and safe for various abdominal organ surgeries. It can be widely applied in elective situations, helping shorten the patient’s hospital stay and speed up recovery [11, 14].

In patients with trauma, not only the abdominal injury itself but also multiple injuries in other parts should be considered. As the metabolic stress of trauma occurs in the patient, the importance of such enteral nutrition adjustment in the patient’s recovery should not be overlooked. However, despite this importance, owing to the diversity of patients, extensive studies on the EEN in patients with trauma have not been conducted.

Therefore, we aimed to compare the factors that affect the timing at which enteral nutrition can be tolerated in patients with BBMI who underwent surgery at a single trauma center. However, the concepts of delayed enteral nutrition and ileus are still difficult to define, and the factors used as indicators of the recovery of gastrointestinal function are controversial. The vague symptoms of the ileus make diagnosis difficult, and its incidence can be assessed differently depending on the definition [15, 16].

Thus, our study analyzed the factors influencing the occurrence of delayed enteral nutrition in patients with early postoperative small bowel obstruction (EPSBO) and the postoperative day of tolerance to solid food and defecation (SF + D), which is a reliable indicator of the patient’s gastrointestinal tract transit [17].

## Methods

### Study population

We reviewed patients who underwent laparotomy for BBMI and were admitted to the Chonnam National University Hospital Regional Trauma Center, Gwangju, Korea, between January 2013 and July 2021. In total, 316 patients were included in this study. Among them, patients who died within 72 h after surgery, those transferred to other hospitals or had a hopeless discharge, and those with a severe traumatic brain injury were excluded. Patients with a Glasgow Coma Scale score of 3–8 points were defined as having severe traumatic brain injury [18].

The degree of abdominal injury was classified according to the organ injury scale (OIS) 2020; operative criteria of the American Association for the Surgery of Trauma grade for blunt small bowel and mesentery injury and other intraabdominal organs were classified by the Abbreviated Injury Scale (AIS) 2015 [19].

**Fig. 1:**
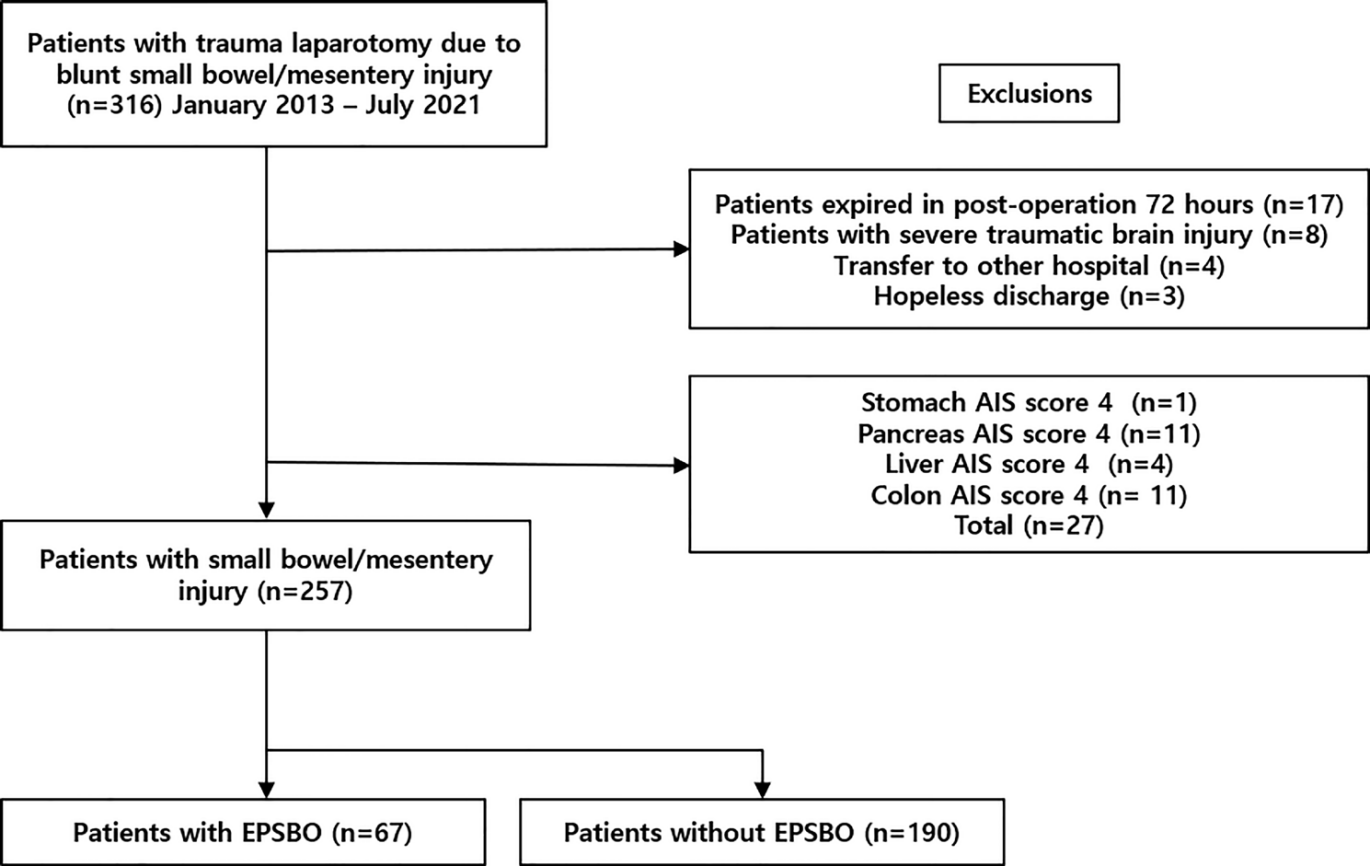
Study Inclusion Flowchart. (AIS, Abbreviated Injury Scale; EPSBO, early postoperative small bowel obstruction)

To reduce the bias for confirming the bowel function recovery of a patient with small bowel/mesentery injury during abdominal surgery, patients with an AIS score for other abdominal organs higher than those for the small bowel or mesentery were excluded (Fig. 1).

However, as the kidney is a retroperitoneal organ, conservative treatment for traumatic kidney injury and its effectiveness are clearly defined even in cases of high-grade renal injury. Moreover, urine extravasation may cause paralytic ileus. Nevertheless, it does not affect other kidney injuries [20]. As paralytic ileus was used as an exclusion criterion in bowel function assessment, patients with higher kidney AIS scores than those for the small bowel or mesentery were included. Patients with severely injured pelvis and lower extremities that could affect gait were also included in the analysis. However, previous studies have reported that pelvic injury can cause ileus in patients [21, 22]. In total, 30 patients had pelvic fracture, and none of the patients with pelvic injury had a pelvic AIS score ≥ 3; thus, all patients were included in the analysis.

### Bowel function assessment

Among 257 patients who were divided according to their small bowel/mesentery OIS scores, EPSBO was used as the criterion for whether the patient’s bowel function recovered and the effect of adhesions [23–25]. In addition to EPSBO, the date of tolerance to SF + D after surgery was confirmed and analyzed.

The diagnosis of EPSBO was classified according to the following criteria: within 30 days after surgery, (1) if flatus passage and bowel function did not return, nausea, vomiting, or abdominal distention occurred, and a regular diet cannot be consumed; or (2) patients had small bowel loops and fluid levels, such as a step ladder sign on plain abdominal radiographic image checked after surgery or a symptomatic image of gas passage retention on abdominal computed tomography (CT) [4, 25].

Among these patients, those who developed these symptoms within 72 h were excluded to avoid the inclusion of patients with paralytic ileus [4, 15, 16, 26].

Gastroparesis is equally important to small bowel function, as we analyzed bowel function based on enteral nutrition tolerance. To exclude gastroparesis, we performed enteral feeding at the duodenal level through a nasogastric feeding tube for patients who could receive enteral nutrition and then included these patients. In addition, some patients had prominent stomach distension, but this was limited to those with stomach AIS scores higher than those for the small bowel. These cases were excluded according to the exclusion criteria. The OIS, AIS 2015, Injury Severity Score (ISS), vital signs and laboratory findings at the time of admission, amount of fluid administered to the patient within 24 h, and amount of transfusion of each component were analyzed.

In addition, to confirm the factors that had an influence after the operation, the aforementioned factors were analyzed by classifying the patients by the surgical method and the number of surgical sites in each group.

Regarding the classification of the operation site, the small bowel operation group was divided into a group with a small bowel OIS score ≥ 2 points and a mesentery OIS score ≥ 1 point. The mesentery operation group had a mesentery OIS score ≥ 2 points and a small bowel OIS score ≥ 1 point. The group with an OIS score ≥ 2 points in both the small bowel and mesentery was referred to as the small bowel and mesentery surgery group.

In addition, patients who had previously undergone an abdominal operation and for whom an anti-adhesive was used were also checked. Sodium hyaluronate and carboxymethyl cellulose components were used as anti-adhesives. The lower extremity AIS score, including that of the pelvis, which can affect the patient’s ambulation, was also compared and analyzed.

### Data analysis

The Pearson chi-squared test and Fisher’s exact test were used for qualitative parameters. Concerning the quantitative parameters, normality of the data was tested using the Shapiro–Wilk test, and Wilcoxon rank-sum test was used to analyze the differences between the groups with and without EPSBO. A *p-*value < 0.05 was used as a criterion for statistical significance.

Correlation analysis was made by Pearson’s product-moment correlation test of various factors related to the postoperative day of tolerance to SF + D was performed.

Multiple logistic regression related to the occurrence of EPSBO was performed using variables with a *p-*value < 0.05, which showed a significant difference between the two groups (EPSBO and non-EPSBO) and the related odds ratio. The Hosmer–Lemeshow test was used for assessment of goodness-of-fit for logistic regression model. A *p-*value > 0.05 was considered a suitable fit for the logistic regression model. The R software, version 3.6 (R Foundation for Statistical Computing, Vienna, Austria), was used for statistical analysis.

## Results

In total, 257 patients were included in this study. A total of 190 and 67 patients were included in the non-EPSBO and EPSBO groups, respectively (Table 1). The proportion of male sex showed a significant difference between the non-EPSBO and EPSBO groups (72.6% vs. 85.1%, respectively; *p* = 0.041). The amount of crystalloid infused in 24 h in the EPSBO group was higher than that in the non-EPSBO group (3,000 vs. 2,400, respectively; *p* = 0.014). There was no significant difference in the use of inotropes or vasopressin between the two groups. The amount of 24-h packed red blood cells (pRBC), fresh frozen plasma (FFP), and platelet concentrate (PC) transfused within 24 h was significantly higher (*p* = 0.015, *p* = 0.022, and *p* = 0.022, respectively) in the EPSBO group that in the non-EPSBO group. There was no difference in the abdominal and extremity AIS scores, but the small intestine OIS (*p* = 0.009) and mesentery OIS (*p* = 0.008) scores were significantly different between the two groups. Based on the postoperative intra-abdominal drain removal date, patients were divided into groups of those who had the drain removed within or over 7 days. The proportion of patients who had the intra-abdominal drain removal within 7 days was significantly higher in the non-EPSBO group than in the EPSBO group (non-EPSBO, 42.1%; EPSBO, 23.9%; *p* = 0.008).

**Table 1:** Characteristics of Patients with Traumatic Small Bowel and Mesentery Injury

	non-EPSBO	EPSBO	p value
	(n = 190)	(n = 67)	
**Age(years)**	55.1 [42.1; 64.1]	55.1 [42.0; 62.0]	
**<65**	144 (75.8)	53 (79.1)	0.581
**>=**65	46 (24.2)	14 (20.9)	
**Sex**			
**Male**	138 (72.6)	57 (85.1)	**0.041**
**Female**	52 (27.4)	10 (14.9)	
**Injury to arrival time(minutes)**	180 [60; 266]	180 [60; 240]	
**<180**	90 (47.4)	30 (44.8)	0.715
**>=**180	100 (52.6)	37 (55.2)	
**Glasgow Coma Scale**	15 [15; 15]	15 [15; 15]	
**<15**	34 (17.9)	13 (19.4)	0.784
**=15**	156 (82.1)	54 (80.6)	
**BMI**	23.6 [21.5; 25.8]	24.2 [22.3; 26.0]	0.861
**Hemoglobin(g/dl)**	12.6 [10.8; 14.2]	12.3 [10.7; 13.9]	0.638
**Base excess(mmol/L)**	-3.8 [-7.4; -0.9]	-2.6 [-5.8; -1.0]	0.093
**pH**	7.4 [7.3; 7.4]	7.4 [7.3; 7.4]	
**<7.35**	80 (42.1)	22 (32.8)	0.182
**>=**7.35	110 (57.9)	45 (67.2)	
**Creatine kinase(IU/L)**	292.5 [158.0; 630.0]	242.5 [140.0; 419.0]	0.795
**Inotropics or vasopressin use**	46 (24.2)	13 (19.4)	0.421
**CPR**	3 (1.6)	1 (1.5)	1.000
**sBP(mmHg)**	100 [80; 110]	100 [80; 110]	
**<90**	55 (29.0)	18 (26.9)	0.745
**>=**90	135 (71.0)	49 (73.1)	
**Crystalloid (cc)**	2400 [1700; 3500]	3000 [2000; 4000]	**0.015**
**24-hr pRBC (units)**	2 [0; 5]	4 [0; 7]	**0.022**
**24-hr FFP (units)**	0 [0; 4]	2 [0; 6]	**0.022**
**24-hr PC (units)**	0 [0; 0]	0 [0; 8]	**0.012**
**Abdomen AIS**	3 [3; 4]	3 [3; 4]	1.000
**Small bowel OIS**	3 [0; 3]	2 [0; 3]	
**<2**	57 (30.0)	32 (47.8)	0.009
**>=**2	133 (70.0)	35 (52.2)	
**Mesentery OIS**	2 [0; 3]	3 [2; 4]	
**<2**	80 (42.1)	16 (23.9)	**0.008**
**>=**2	110 (57.9)	51 (76.1)	
**Extremity AIS**	0 [0; 2]	0 [0; 2]	
**<2**	140 (73.7)	45 (67.2)	0.307
**>=**2	50 (26.3)	22 (32.8)	
**ISS**	16 [9; 20]	16 [9; 22]	
**minor 1–8**	11 (5.8)	4 (6.0)	0.585
**moderate 9–15**	77 (40.5)	25 (37.3)	
**serious 16–24**	78 (41.1)	25 (37.3)	
**severe 25–49**	24 (12.6)	13 (19.4)	
**Post operative drain removal date(days)**	7 [5; 9]	8 [7; 10]	
**<7**	80 (42.1)	16 (23.9)	**0.008**
**>=**7	110 (57.9)	51 (76.1)	
**ICU stay(days)**	3 [2; 6]	4 [2; 6]	0.238

Categorical variables are expressed as numbers (%), and continuous variables are presented as medians [first and third quartiles]. pH, percentage of hydrogen ions; CPR, cardiopulmonary resuscitation; sBP, systolic blood pressure; AIS, Abbreviated Injury Scale; OIS, Organ Injury Scale; ISS, Injury Severity Score; ICU, intensive care unit; BMI, body mass index; pRBC, packed red blood cells; FFP, fresh frozen plasma; PC, platelet concentrate

The surgical variables were analyzed between the EPSBO and non-EPSBO groups. A previous history of abdominal operation showed no significant difference between the two groups. The injury site was differentiated by the small bowel, mesentery, small bowel, and mesentery, which showed statistical differences between the two groups (small bowel, 41.9% vs. 23.9%, mesentery 29.8% vs. 47.8%; small bowel and mesentery, 28.3% vs. 28.4%; *p* = 0.011). The type of surgery, number of sutures or anastomosis sites, and anti-adhesive material used were not significantly different between the two groups. In total, 27 patients underwent damage control surgery. Of the 27 patients, 22 underwent damage control surgery (DCS) due to coagulopathy, and three underwent DCS because they needed bowel reassessment. Additionally, two patients were confirmed to have undergone damage control surgery for bowel edema and extra abdominal causes. There was no statistically significant difference in the incidence of EPSBO in the DCS group. Three out of five patients who did not undergo DCS for coagulopathy indication were included in the EPSBO group (Table 2).

**Table 2:** Analysis of Surgical Variables in Patients with Blunt Small Bowel/Mesenteric Injury with/without EPSBO.

	non-EPSBO	EPSBO	p
	(N = 190)	(N = 67)	
**Previous abdomen operation history**			
**No**	157 (82.6)	60 (89.6)	0.179
**Yes**	33 (17.4)	7 (10.4)	
**Operation time(minutes)**	115 [94; 145]	115 [94; 140]	
**<180**	167 (87.9)	58 (86.6)	0.777
**>=**180	23 (12.1)	9 (13.4)	
**Injury site**			
**Small bowel**	56 (29.5)	32 (47.8)	**0.010**
**Mesentery**	54 (28.4)	19 (28.3)	
**Small bowel and mesentery**	80 (42.1)	16 (23.9)	
**Small bowel segmental resection**	100 (52.6)	28 (41.8)	0.127
**Small bowel primary repair**	59 (31.0)	16 (23.9)	0.267
**Small bowel serosal repair**	4 (2.1)	4 (6.0)	0.211
**Mesentery suture repair**	46 (24.2)	20 (29.9)	0.364
**Damage control surgery**	17 (9.0)	10 (14.9)	0.170
**Small bowel anastomosis and suture sites**			
**0**	30 (15.8)	17 (25.4)	0.218
**1**	132 (69.5)	41 (61.2)	
**> 2**	28 (14.7)	9 (13.4)	
**Anti-adhesive use**	102 (53.7)	31 (46.3)	0.296

Categorical variables are expressed as numbers (%), and continuous variables are presented as medians [first and third quartiles]

EPSBO, early postoperative small bowel obstruction

Five patients underwent revision surgeries for EPSBO. Two patients underwent segmental resection of the small intestine for focal intestinal ischemia, and three underwent adhesiolysis.

Two patients underwent surgeries at 1 month after the first operation. Both patients underwent adhesiolysis. Two patients were readmitted at > 3 months after discharge due to obstructive ileus and underwent segmental resection. One patient underwent adhesiolysis and wound revision after 3 weeks of the initial surgery. All the other 62 patients were managed conservatively, and no other adverse events were associated with bowel function occurred.

When analyzing each injury site and comparing the characteristics and the frequency of EPSBO occurrence, a statistically significant difference was observed.

Among the OIS and AIS scores of all injury sites, patients with a small bowel OIS score of 3 points constituted the largest proportion (76% in the small bowel injury group and 75.3% in the small bowel and mesentery injury group).

For the mesentery, cases with OIS scores of 2, 3 and 4 points showed a generally even distribution (OIS scores of 2, 3, and 4 points: 28.1%, 34.8%, and 37.1%, respectively, in the mesentery injury group; and OIS scores of 2, 3, and 4 points: 35.6%, 31.5%, and 32.9%, respectively, in the small bowel and mesentery injury group). Therefore, the small bowel injury group showed a result that seemed to increase the EPSBO incidence more specifically when classified into injury site groups (Table 3.)

**Table 3:** Analysis of Injury Sites in Patients with Blunt Small Bowel/Mesenteric Injury with/without Early Postoperative Small Bowel Obstruction (EPSBO)

Injury sites	Small bowel (n = 96)	Mesentery (n = 89)	Small bowel and mesentery(n = 73)	*p*
**Small bowel OIS**				< 0.001
<1	0 ( 0.0%)	89 (100.0%)	1 ( 1.4%)	
2	4 ( 4.2%)	0 ( 0.0%)	5 ( 6.8%)	
3	73 (76.0%)	0 ( 0.0%)	55 (75.3%)	
4	19 (19.8%)	0 ( 0.0%)	12 (16.4%)	
**Mesentery OIS**				< 0.001
<1	96 (100.0%)	0 ( 0.0%)	0 ( 0.0%)	
2	0 ( 0.0%)	25 (28.1%)	26 (35.6%)	
3	0 ( 0.0%)	31 (34.8%)	23 (31.5%)	
4	0 ( 0.0%)	33 (37.1%)	24 (32.9%)	
**ISS**	15.4 ± 7.2	16.9 ± 8.2	15.6 ± 7.4	0.736
**EPSBO**	80 (83.3%)	57 (64.0%)	54 (74.0%)	0.011
**Non-EPSBO**	16 (16.7%)	32 (36.0%)	19 (26.0%)	

Categorical variables are expressed as numbers (%)

OIS, Organ Injury Scale; ISS, injury severity score; EPSBO, early postoperative small bowel obstruction

Logistic regression analysis was performed between the EPSBO and non-EPSBO groups using variables with *p-*values < 0.05. The esentery OIS score (adjusted odds ratio [AOR]: 2.1; 95% confidence interval [CI]: 1.07–4.12; *p* = 0.031) and the small bowel OIS score (AOR: 0.52; 95% CI: 0.28–0.95; *p* = 0.035) were identified as the factors related to EPSBO (Table 4).

**Table 4:** Logistic Regression Model for Odds Ratio of Patients with Early Postoperative Small Bowel Obstruction

	Odds ratio	*P*-value	95% CI	
**Sex**	0.478756	0.062	0.221	1.037
**Crystalloid**	1.000074	0.515	0.999	1.000
**24-h pRBC**	0.98767	0.862	0.858692	1.13602
**24-h FFP**	0.951535	0.549	0.808943	1.119261
**24-h PC**	1.073206	0.079	0.991895	1.161182
**Mesentery OIS**	2.101753	0.031	1.071886	4.121114
**Postoperative drain removal day**	1.854631	0.071	0.949306	3.62334
**Small bowel OIS**	0.52391	0.035	0.287284	0.955438
**Injury site**	1.46	0.297	0.711	2.940

pRBC, packed red blood cell; FFP, fresh frozen plasma; PC, platelet concentrate; ; OIS, Organ Injury Scale; CI, confidence interval

**Fig. 2:**
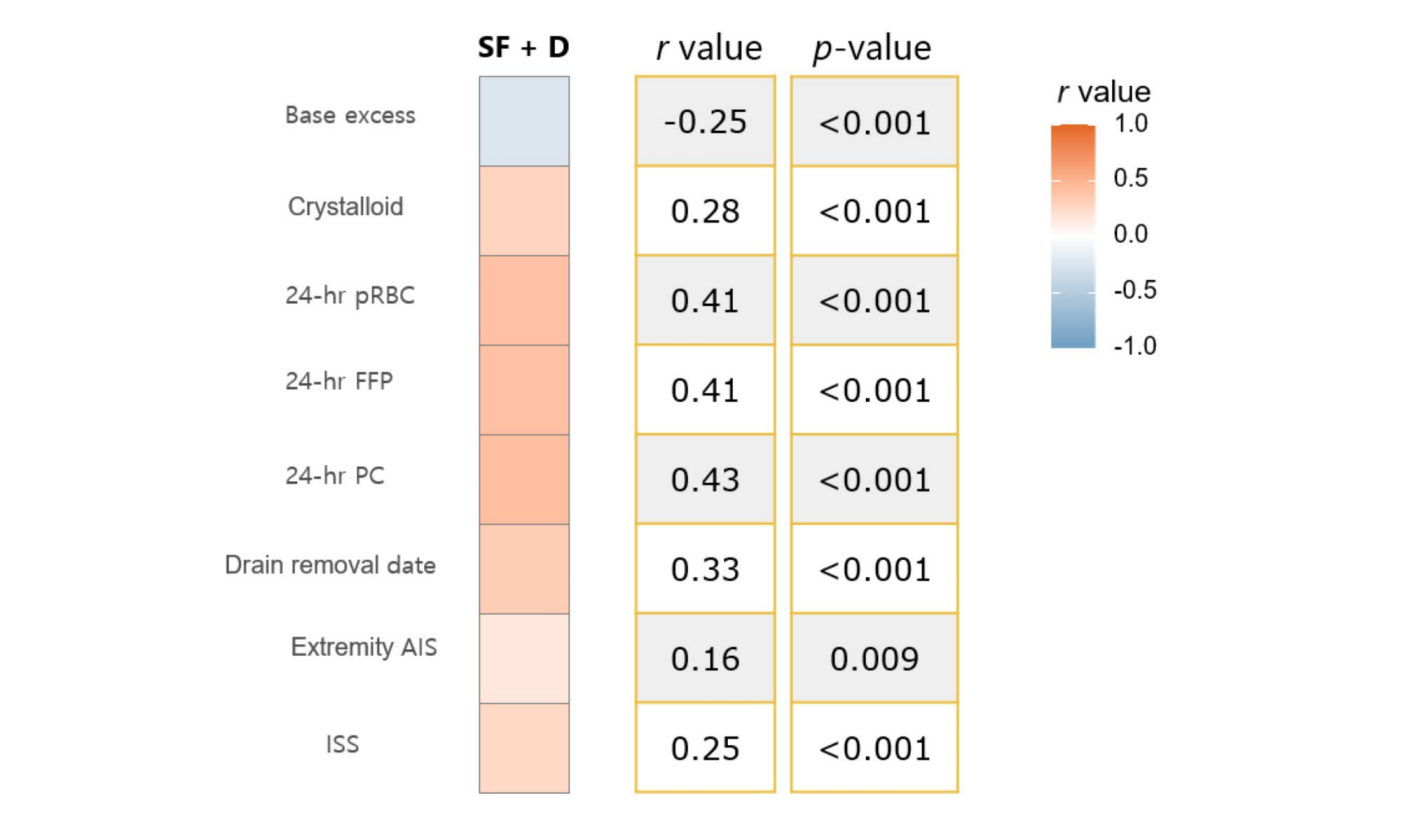
The Heatmap of Correlation Coefficients by Pearson’s Product-Moment Correlation. (Correlation analysis between the factors associated with small bowel/mesenteric injury due to blunt trauma and postoperative day until patients tolerated SF + D. SF + D solid food and defecation, pRBC, packed red blood cells; FFP, fresh frozen plasma; PC, platelet concentrate; AIS, Abbreviated Injury Scale; ISS, Injury Severity Score)

Among these patients, five who underwent re-operation with EPSBO were excluded. We correlated the postoperative day of tolerance to SF + D and several factors in the remaining patients. The amount of crystalloid infused within 24 h; the amount of pRBC, FFP, and PC transfused within 24 h; the time of drain removal; ISS; and extremity AIS score showed a correlation with the day of tolerance to SF + D (Fig. 2).

## Discussion

Early postoperative enteral nutrition is vital for patient recovery. Prolonged fasting may adversely affect the functional recovery of the patients’ gut homeostasis and barrier function [27]. Therefore, EEN is a part of the ERAS protocol and is applied in most surgeries, including gastrectomy, liver, colorectal and pancreatic resections, pelvic surgery, and even transplantation surgery [13, 28–31].

However, among these ERAS protocols, the concept of EEN is difficult to apply to emergency surgery rather than elective surgery, especially in patients with traumatic injuries. In fact, there have been several studies that aimed to apply enhanced recovery to emergency laparotomy. Most of the studies were limited to specific diseases (i.e., peptic ulcer disease, colectomy) [32, 33] and the number of studies related to emergencies in the small bowel was very limited. In a recent study on laparotomy, patients with trauma were excluded from the study [34, 35].

Among patients with trauma, there are a few cases, in which abdominal laparotomy is the only treatment due to injury. Most of the injuries caused by blunt trauma are often accompanied by multiple traumas in other areas, and there are also many cases where the treatment involves multiple departments through multimodality and requires intensive treatment. Because of these circumstances, it is difficult to recover the patient’s bowel motility and function, and it is more challenging to apply EEN [4, 36, 37]. In addition, these patients not only have bowel injuries but are also in shock or in a critically ill state, making treatment difficult. The importance of dietary adjustments should not be overlooked, as delayed feeding for various reasons may delay other necessary treatments. As early EEN has been recommended for patients after surgery and for critically ill adult patients, the factors that can influence the initiation and influence of active EEN in patients with trauma should be considered. However, extensive studies on enteral nutrition tolerance in patients with trauma have not been conducted to date. There have been attempts related to ERAS implementation in patients with penetrating abdominal trauma, but the cases of patients with traumatic injury due to blunt force are different and distinct [36]. For the classification of the organ injury scale, small bowel injuries are divided into blunt and penetrating [19]. When the abdominal wall is injured by a blunt mechanism (such as seat belt, crush, fall, handlebar injuries), the transmitted impact can increase the gastrointestinal tract intraluminal pressure or cause a burst injury to the mesentery itself [1, 6].

In the study by Jianyi et al., the patients with abdominal trauma in the group that performed EEN within 72 h of hospitalization in the intensive care unit had a positive effect on enteral feeding tolerance and recovery than those in the group that did not receive EEN [38]. Moreover, the patients with abdominal trauma in the group that performed EEN within 72 h of hospitalization in the intensive care unit had a more positive effect on enteral feeding tolerance and recovery than those in the group that did not receive EEN. However, patients who did not undergo surgery and those with multiple intra-abdominal organ injuries were also included. In addition, there were limitations in the characteristics between the two groups, such as differences in the injured organ, injury mechanism, and laparotomy [38]. Recently, as the patient’s gut has been recognized to play an important role in immunologic function, the degree of metabolic stress caused by multiple traumas itself is large; thus, blood glucose control and stress-related catabolism in the patient need to be controlled in most situations. Therefore, to prevent progression to systemic inflammatory response syndrome or multi-organ failure due to bacterial translocation, EEN minimizes gut injury and can be expected to play an immunomodulatory role [39].

Kang et al. conducted a study at our chonnam national university hospital regional trauma center, Gwangju, Korea, in 2018 and reported that male sex, use of intraoperative crystalloid, and OIS score for mesenteric injury were significant independent risk factors for incidence of EPSBO in patients undergoing laparotomy for trauma. However, the analysis included patients who underwent traumatic laparotomy of the general abdominal area, such as liver, pancreas, and spleen injuries, by blunt and penetrating injury mechanisms. Therefore, it is difficult to apply the findings to a specific trauma disease [4].

However, in this study, the inclusion criteria were limited to abdominal injuries by blunt force, and only those who underwent emergency operations were selected and studied. In addition, we focused on the small bowel/mesenteric injury itself, excluding surgeries for injuries of the liver, pancreas, or colon. In addition, for the difficulty in diagnosing the patient’s postoperative ileus, the time point at which successful solid diet tolerance and defecation occurred was analyzed to determine the variables that could affect the patient more clearly.

In this study, the factors that affected the incidence of EPSBO in patients with traumatic small bowel and mesentery injury were as follows: male sex, small bowel and mesentery OIS scores, amount of crystalloid and blood transfusion, and the postoperative drain removal date. A higher mesentery OIS score was associated with a higher EPSBO incidence, whereas the small bowel OIS score was not related to an increased incidence of EPSBO (AOR: 0.52; 95% CI: 0.28–0.95; *p* = 0.035).

The recovery of bowel function was delayed in patients with substantial mesenteric injury (high OIS score). Previously, there have been a few studies on how mesenteric injuries affect the ileus [8]. The presumed cause is that the patient’s mesenteric injury may cause temporary intestinal ischemia depending on the extent of vascular injury, and it may take some time to restore the blood supply and normal gut function due to the occurrence of a hematoma. In fact, in several case reports, delayed ischemic stenosis due to blunt mesenteric injury has been reported. In these cases, stenosis or decreased motility was confirmed through surgery, and even if the patient did not have direct bowel injury, bowel function impairment could occur through mesenteric injury [40, 41].

Small bowel OIS (AOR: 0.52; 95% CI: 0.28–0.95; *p* = 0.035) scores were identified as the factors not related to EPSBO. However, on sub-analysis by injury site, injury of the small bowel site showed a result that seemed to increase the EPSBO incidence more specifically as an injury site value. This result seems to indicate that when a higher grade of injury of the small intestine (OIS > 4 points) occurs, the damaged small bowel can be broadly resected. However, when a moderate grade injury occurs (OIS score of 3 points; small bowel injury combined with transection and minimal contamination) in the small bowel, surgical intervention of the injury site might be limited, and the residual injury (hematoma and temporary injury) might cause EPSBO [19]. In fact, patients with injuries of the small bowel with an OIS score of 3 points constituted the largest proportion (76%) in the small bowel injury group.

In addition, according to the CT analysis in patients with abdominal injury due to BBMI, when a mesenteric injury was observed on CT, spontaneous resolution compensation or secondary perforation due to delayed ischemia may be observed depending on the degree of damage [42]. Therefore, in patients with a mesenteric injury due to contusion, it may take some time for the patient’s bowel function to return depending on the degree of spontaneous resolution of the remaining part even if surgical resection is appropriately performed according to the degree of injury in the preoperative CT image or surgical field. However, in cases of small bowel injury, if the mesenteric injury is small, relatively few factors can delay solid food intake after surgery because the operation is performed on the direct injury site. Thus, the diet should not be overlooked in patients with mesenteric injury, and further studies are needed to examine the relationship between the patient’s classification of the mesentery and small bowel OIS scores and the timing of enteral nutrition tolerance.

Small bowel and mesenteric injuries are difficult to distinguish clearly in clinical settings. Therefore, to reduce such bias in this study, injuries with OIS scores between 0 and 1 were integrated and analyzed [19, 42]. In addition, by analyzing each operation, the surgical method and the number of surgical sites were subdivided to evaluate for additional bias or other influencing factors.

The amount of crystalloid infused in 24 h was higher in the EPSBO than in the non-EPSBO group (3,000 vs. 2,400, respectively; *p* = 0.015). In most patients, balanced fluid volumes are recommended. Several studies have revealed that the amount of crystalloid infused is crucial in bowel recovery [43]. Optimal fluid resuscitation can regenerate circulation of the mesentery and small bowel. However, the optimal fluid amount in patients with trauma is difficult to estimate. Traumatic shock state and initial excessive fluid resuscitation often result in increased intestinal permeability and can even cause ascites. Moreover, during restrictive fluid administration, decreased blood flow to the mesentery and small bowel can further lead to tissue hypoxia and tissue edema [44]. It is difficult to know whether the appropriate amount of fluid is administered to patients with trauma. However, our study showed that administering a higher amount of fluid to the patients could induce EPSBO.

Massive transfusion of blood products is the main cause of abdominal compartment syndrome and interstitial edema. Our study showed that the amount of transfused blood components within 24 h was significantly higher (*p* = 0.015, *p* = 0.022, and *p* = 0.022, respectively) in the EPSBO group. There were 35, 12, and 23 patients who received massive transfusion (more than 10 packs of pRBC) in total, in the EPSBO group, and in the non-EPSBO group, respectively [45].

Intra-abdominal infection might play role in EPSBO and EN tolerance [46]. In addition to the actual bowel injury OIS, we investigated the surgical variables and tried to confirm related associations of intra-abdominal infection and EPSBO in our data, but no meaningful results were derived. In addition, we tried to confirm the relationship with infection by examining the indications of damage control patients, but again, there was no specificity.

The factors thought to affect the patient’s bowel function, such as the ISS score, use of inotropes/vasopressors, damage control surgery, and history of previous abdominal surgery, did not significantly affect the recovery from EPSBO or timing of enteral nutrition tolerance in this study.

Our study had some limitations. This was a single-center study with a retrospective design. In addition, the amount of opioids used after surgery was not included in the study as a factor that could affect bowel function after surgery. To analyze the importance of ambulation, the pelvis and lower extremity AIS scores were analyzed together; there was no difference between the two groups, but the patient’s performance score was not analyzed. Furthermore, intraabdominal infection or microbiota’s role in patients with trauma was not clearly clarified. A large-scale, multicenter, follow-up study to examine whether there is an effect on the long-term follow-up of patients should be conducted.

## Conclusion

The factors that affected the incidence of EPSBO in patients with BBMI were male sex as well as small bowel and mesentery OIS scores. Higher mesentery OIS scores were associated with higher EPSBO incidence, whereas the small bowel OIS score was not related to the incidence of EPSBO. The amount of crystalloid infused within 24 h; the amount of transfusion of pRBC, FFP, and PC; the time of drain removal; ISS; and extremity AIS were related to the postoperative day when patients tolerated SF + D.

This suggests that mesenteric injury has a greater impact on EPSBO than small bowel injury. However, further research is needed to determine whether the mesentery OIS score should be considered as an indicator for EEN in patients with BBMI.

## Data Availability

The data that support the findings of this study are available on request from the corresponding author. The data are not publicly available due to privacy or ethical restrictions.

## Abbreviations

AIS: abbreviated injury scale
BBMI: blunt bowel and/or mesenteric injury
CT: computed tomography
DCS: damage control surgery
EEN: early enteral nutrition
EPSBO: early postoperative small bowel obstruction
ERAS: enhanced recovery after surgery
FFP: fresh frozen plasma
ISS: Injury Severity Score
OIS: organ injury scale
PC: platelet concentrate
pRBC: packed red blood cells
SF + D: solid food and defecation
